# Branched-chain amino acid metabolism in cancer

**DOI:** 10.1097/MCO.0000000000000430

**Published:** 2017-12-01

**Authors:** Elitsa A. Ananieva, Adam C. Wilkinson

**Affiliations:** a Department of Biochemistry and Nutrition, Des Moines University, Des Moines, Iowa; b Department of Genetics, Institute for Stem Cell Biology and Regenerative Medicine, Stanford University School of Medicine, Lorry I. Lokey Stem Cell Research Building, Stanford, California, USA

**Keywords:** branched-chain amino acids, branched-chain aminotransferase 1, branched-chain aminotransferase 2, cancer

## Abstract

**Purpose of review:**

The current review aims to provide an update on the recent biomedical interest in oncogenic branched-chain amino acid (BCAA) metabolism, and discusses the advantages of using BCAAs and expression of BCAA-related enzymes in the treatment and diagnosis of cancers.

**Recent findings:**

An accumulating body of evidence demonstrates that BCAAs are essential nutrients for cancer growth and are used by tumors in various biosynthetic pathways and as a source of energy. In addition, BCAA metabolic enzymes, such as the cytosolic branched-chain aminotransferase 1 (BCAT1) and mitochondrial branched-chain aminotransferase 2, have emerged as useful prognostic cancer markers. BCAT1 expression commonly correlates with more aggressive cancer growth and progression, and has attracted substantial scientific attention in the past few years. These studies have found the consequences of BCAT1 disruption to be heterogeneous; not all cancers share the same requirements for BCAA metabolites and the function of BCAT1 appears to vary between cancer types.

**Summary:**

Both oncogenic mutations and cancer tissue-of-origin influence BCAA metabolism and expression of BCAA-associated metabolic enzymes. These new discoveries need to be taken into consideration during the development of new cancer therapies that target BCAA metabolism.

## INTRODUCTION

Cancer cells have unlimited potential to divide and sustain growth. This process is dependent on acquiring essential nutrients from the tumor microenvironment, which are used to maintain biomass and survival, even under conditions of poor nutrient and oxygen availability [1,2^▪^,^3^]. The metabolic flexibility of cancer cells is determined by their ability to reprogram anabolic and catabolic pathways, through altering gene expression programs as well as intercellular interactions within the tumor microenvironment [4].

The process of oncogenesis is dependent on amino acids, the building blocks for protein synthesis, and a source of energy and metabolites [3]. Many cancer types overexpress enzymes that function to degrade amino acids, which not only provide cellular energy and metabolites for anabolic processes but also serve as mechanisms of immune evasion by cancer cells [2^▪^,^5^]. For example, tumor overexpression of indoleamine-2,3, dioxygenase and arginase depletes the tumor microenvironment of tryptophan and arginine, respectively, which is beneficial for tumor growth but also suppresses local cytotoxic T-cell proliferation [6–8]. Thus, by using amino acid degrading enzymes as immunosuppressive factors, tumors increase their ability to survive.

**FIGURE 1:**
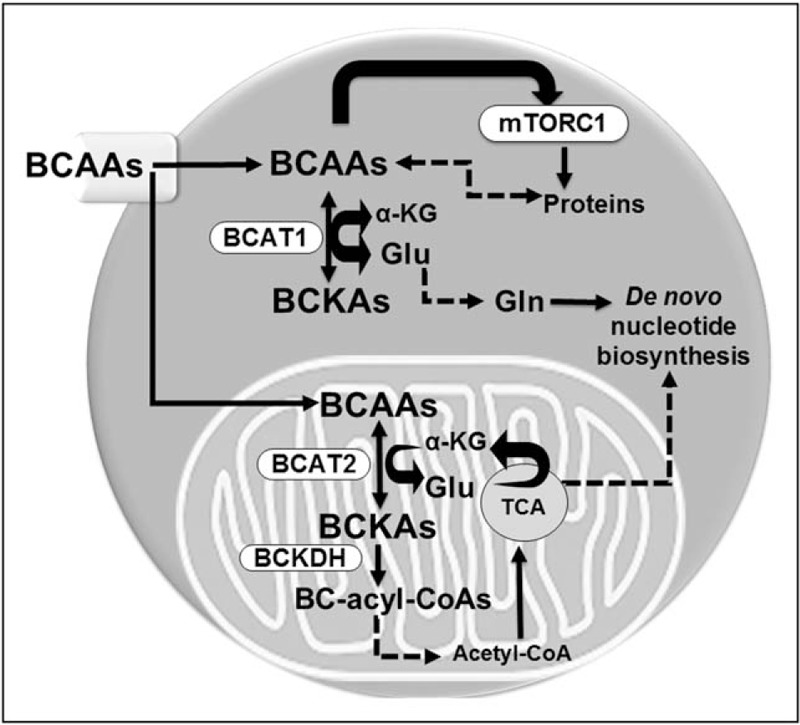
A model of branched-chain amino acid metabolism in cancer. As essential amino acids, cancer cells must obtain branched-chain amino acids from the tumor microenvironment or from protein degradation. Branched-chain amino acids are thought to play several roles in cancer cells: activate complex 1 of the mammalian target of rapamycin signaling, which stimulates protein translation, growth, and survival; serve as building blocks in protein synthesis; be metabolized into branched-chain α-keto acids in the cytosol (by branched-chain aminotransferase 1) and/or mitochondria (by branched-chain aminotransferase 2), a process involving conversion of α-ketoglutarate to glutamate; serve as indirect source of nitrogen for nucleotide (and nonessential amino acid) biosynthesis via the glutamate–glutamine axis; and become further catabolized to yield acetyl-CoA and succinyl-CoA (not shown) that feed into the cycle of tricarboxylic acids cycle and can contribute to energy production. Note that in some cancers (such as chronic myeloid leukemia), branched-chain aminotransferase 1 is proposed to convert branched-chain α-keto acids back to branched-chain amino acids. BCAA, branched-chain amino acid; BCKA, branched-chain α-keto acid; BCKDH, branched-chain keto acid dehydrogenase; BC-acyl-CoAs, branched-chain acyl-CoAs; α-KG, α-ketoglutarate; TCA, cycle of tricarboxylic acids; mTORC1, complex 1 of the mammalian target of rapamycin.

In addition to arginine and tryptophan, tumors also preferentially uptake the branched-chain amino acids (BCAAs) leucine, isoleucine, and valine [5]. BCAAs can be used for protein synthesis or oxidized for energy purposes by tumors. BCAAs are essential amino acids; tumors must rely on dietary BCAA intake and their release from protein degradation [9] (Fig. 1). In recent years, it has become evident that the enzymes catalyzing the first step in BCAA degradation are overexpressed in many cancers [10,11,12^▪^]. These are the cytosolic [branched-chain aminotransferase 1 (BCAT1)] and mitochondrial [branched-chain aminotransferase 2 (BCAT2)] branched-chain aminotransferases, which convert BCAAs into their corresponding branched-chain α-keto acids by transferring the amino group onto α-ketoglutarate and thereby generating glutamate [13]. Of the two enzymes, BCAT1 is the major isoform implicated in cancer growth and has been proposed as a prognostic cancer cell marker [5,10,14–17,18^▪▪^]. The role of BCAT1 in cancer progression has become an intriguing but challenging topic to understand, with several different functions in tumor growth having been proposed [12^▪^,^18^^▪▪^]. In this review, we summarize the latest discoveries on the utility of BCAT1 expression as a prognostic cancer cell marker and the recent mechanistic insights into how BCAT1 contributes to the metabolic reprograming of cancer cells. Next, we address the most recent understanding of the role of BCAA metabolism in cancer growth and progression. Lastly, we discuss current and future opportunities to clinically target BCAA metabolism in the context of cancer therapies. 

**Box 1:**
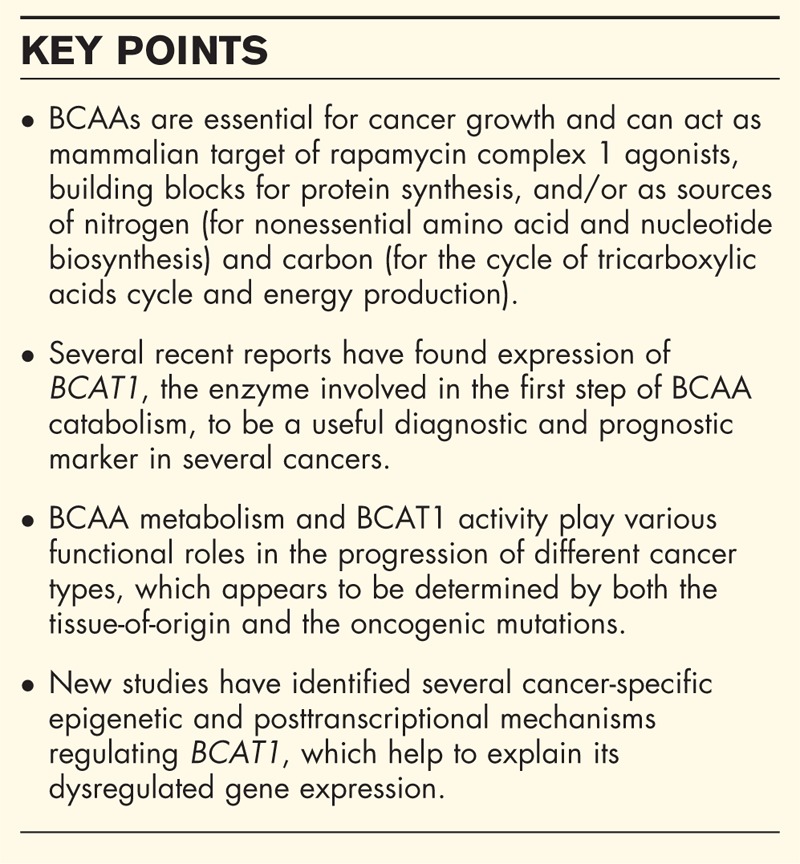
no caption available

## BRANCHED-CHAIN AMINOTRANSFERASE 1 IS A PROGNOSTIC CANCER MARKER AND AN ATTRACTIVE TARGET FOR CANCER THERAPIES

The role of BCAT1 in cancer development was largely overlooked until recently. It was not until 2013, when Tonjes *et al.*[10] reported overexpression of BCAT1 in gliomas, that the scientific community became interested in this metabolic enzyme and its potential in cancer therapy. Since 2013, scientific knowledge about BCAT1 in cancer has been steadily accumulating with an average of seven publications/year (per ‘PubMed’ search). Current knowledge indicates that most cancer types express high levels of BCAT1 [5,14,17]. By contrast, BCAT1 expression in healthy humans is mainly limited to the nervous system and gonadal tissues [5], as well as activated T lymphocytes [13] and macrophages [19]. The cancer-specific expression of *BCAT1* makes this gene an attractive target for therapeutic intervention. However, the biological functions of BCAT1 in cancer are not well understood, and recent evidence suggests it may be dependent on the cancer tissue-of-origin [9,12^▪^].

BCAT1 expression in glioblastoma tumors is specific to those carrying wild-type isocitrate dehydrogenase 1 and 2 (IDH1 and IDH2) [10]. Mutations in either IDH1 or IDH2, commonly seen in glioblastomas, contribute to downregulation of BCAT1 through DNA methylation of the BCAT1 promoter and the corresponding epigenetic silencing of *BCAT1*[10] (Table 1). Mutations in IDH1/2 are common in gliomas and acute myeloid leukemia (AML), whereas solid tumors rarely harbor IDH mutations [10,15,20,21]. Significantly, BCAT1 was recently found to be highly expressed in AML, where it contributed to growth *in vitro*[18^▪▪^]. On the other hand, an inverse relationship between BCAT1 and IDH1/2 was found in epithelial ovarian cancer (EOC), where BCAT1 silencing suppressed the expression of *IDH1/2* genes [15]. Within the cycle of tricarboxylic acids (TCA) cycle, wild-type IDH enzymes convert isocitrate into α-ketoglutarate, whereas mutant IDH enzymes convert isocitrate into hydroxyglutarate [10]. Given α-ketoglutarate is used by BCAT1 for BCAA transamination, this points toward a possible metabolic link between IDH1/2 and BCAT1. Resulting perturbations to TCA cycle-associated metabolites and energy production may have contributed to the accelerated cellular proliferation, migration, and invasion observed in EOC [15] (Table 1).

Most, but not all, reports indicate that BCAT1 overexpression correlates with enhanced cancer growth, whereas suppression of BCAT1 limits proliferation. For example, suppression of BCAT1 in U-87MG, a human primary glioblastoma cell line, produced smaller tumors in mice [10]. Similarly, when *Bcat1*-null nonsmall lung carcinoma (NSCLC) cells were implanted subcutaneously in mice, these cells displayed impaired tumor-forming ability [12^▪^]. However, when mice were injected with SKOV3 ovarian carcinoma cells with suppressed BCAT1 expression, tumor burden was not alleviated, although survival rates were significantly increased as compared with control animals [15] (Table 1). Likewise, suppression of BCAT1 in pancreatic ductal adenocarcinoma (PDAC) did not lead to a reduction in tumor growth, and patients with PDAC expressed low levels of tumor BCAT1 and displayed increased plasma BCAAs levels [12^▪^]. Thus, not all cancer types express high levels of BCAT1 and suppression of BCAT1 does not always correspond to a decrease in tumor size.

In several tumor types, the epigenetic dysregulation of *BCAT1* expression has been elucidated. The best described epigenetic mechanism involves mutated IDH1/2, as discussed above [10]. However, another epigenetic mechanism, involving the disruptor of telomeric silencing 1-like (DOT1L) histone methyltransferase, was recently proposed [22]. In contrast to *IDH1/2* mutations, DOT1L activates *BCAT1* gene expression through histone H3K79 methylation of the coding region, but not the promoter, of *BCAT1*[22]. In leukemias, driven by genetic mutation of the *mixed lineage leukemia 1* (*MLL1*) gene, DOT1L maintains an open chromatin state and gene transcription. DOT1L also forms part of the elongation assisting protein complex, along with positive transcription elongation factor b, among others, which is recruited by oncogenic MLL1 fusion proteins (such as MLL-AF9) to stimulate RNA Pol II gene transcription [23]. In addition, DOT1L can cooperate with c-Myc and p300 to enhance transcription [24]. *BCAT1* has also been described as a downstream target of c-Myc in many cancers, including ovarian and liver cancer [5,15,17]. Thus, it is intriguing to speculate that at least one mechanism of *BCAT1* upregulation in cancer may involve cooperation between DOT1L and c-Myc.

Most recently, a new posttranscriptional regulator of *BCAT1* expression was identified in chronic myeloid leukemia (CML), the musashi RNA binding protein 2 (MSI2) [18^▪▪^]. MSI2 and BCAT1 are coexpressed in CML blast crisis, and a physical interaction between MSI2 protein and *BCAT1* mRNA was identified, suggesting that BCAT1 expression in CML is MSI2-dependent. Moreover, the MSI2–BCAT1 axis was proposed as an important mechanism in driving cancer progression in CML [18^▪▪^] (Table 1).

Taken together, these studies point toward a role of BCAT1 as a prognostic cancer marker, although the mechanisms of *BCAT1* gene dysregulation appear to differ between cancer types. In addition, these data suggest inhibition of BCAT1 activity may be a useful therapeutic strategy in the treatment of several cancers.

## REPROGRAMING OF BRANCHED-CHAIN AMINO ACID METABOLISM TO ACTIVATE MAMMALIAN TARGET OF RAPAMYCIN SIGNALING IN CANCER

The BCAA leucine is a well described mammalian target of rapamycin (mTOR) agonist [13], and Sestrin2 was recently identified as a direct intracellular leucine sensor and mTOR complex 1 (mTORC1) regulator [25^▪▪^,^26^]. Many cancers rely on constitutive mTOR activity to maintain cellular growth and proliferation [27]. Recent reports have linked BCAT1 expression to mTOR activity in several cancers, although different mechanisms have been proposed [16,18^▪▪^]. Hattori *et al.*[18^▪▪^] demonstrated that BCAT1 was overexpressed in CML blast crisis. In this context, rather than deaminating BCAAs to BCKAs, BCAT1 overexpression resulted in increased intracellular concentrations of BCAAs through BCKA amination. Reduced BCAT1 expression (or activity through pharmacological inhibition) resulted in reduced mTORC1 activity, presumably through reduced intracellular BCAA concentrations. Importantly from a therapeutic standpoint, knockdown of BCAT1 in a mouse model of CML improved survival, while use of the BCAT1 inhibitor gabapentin suppressed colony formation of human patient CML [18^▪▪^].

Zhang and Han [16] also found BCAT1 expression promoted mTOR activity, but in the context of breast cancer cells. The authors found that BCAAs were increased in patients with breast cancer (compared with healthy controls), in both peripheral blood serum and cancer tissue, and BCAT1 was also overexpressed. BCAT1 expression also contributed to the growth of breast cancer cell lines and appeared to act through mTORC1 activity (Table 1). However, downstream BCAA catabolic enzymes were also overexpressed in breast cancer cells [16], suggesting catabolism of BCKAs into the TCA cycle may also play a role in this context. Significantly, recent metabolic analysis of *BRCA1*-mutant breast epithelial cells also identified increased BCAA concentrations, suggesting increased BCAA concentrations via reprogramed metabolism may be an early event in *BRCA1*-cancer development [28].

Further mechanistic insight into the role of BCAT1 overexpression in breast cancer was recently identified by Thewes *et al.*[29]. In ERα-negative breast cancer, BCAT1 indirectly regulated the cell cycle regulator retinoblastoma protein through the cell cycle inhibitor p27^Kip1^. Here, BCAT1 controlled cell cycle progression, sustaining breast cancer proliferation. By contrast, Takegoshi *et al.*[30] suggested that BCAAs prevented development of hepatocellular carcinoma in mice models of nonalcoholic steatohepatitis. As in the studies described above, BCAAs appeared to act via mTORC1. These data suggest BCAAs have cancer-specific functions and that in certain contexts, BCAAs may even suppress cancer development.

Combined, these recent findings highlight the importance of BCAT1 and BCAA metabolism in reprograming cancer metabolism via mTORC1, with profound consequences on cell cycle and cancer progression.

## BRANCHED-CHAIN AMINO ACIDS SUPPORT THE CANCER ENERGETIC AND BIOSYNTHETIC DEMANDS

BCAAs play an important role in energy homeostasis and nutrient signaling as well as nitrogen balance [31,32]. Several recent studies have found BCAA metabolism to be an important ‘module’ within cancer metabolism, but appear to drive cancer progression by diverse mechanisms [9,11,12^▪^]. For example, transamination of BCAAs leads to formation of glutamate, which can be used for biosynthesis of other nonessential amino acids such as glutamine, or recycled to α-ketoglutarate via other aminotransferases [33,34]. In NSCLC tumors, high glutamate and glutamine concentrations correlated with an increased expression of BCAT1 and higher rates of BCAA uptake [12^▪^]. Similarly, knockdown of BCAT1 expression (or pharmacological BCAT1 inhibition) in glioblastoma cells reduced the formation of glutamate [10]. However, not all cancers produced high levels of glutamate in response to overexpression of BCAT1. As described above, in CML blast crisis, high expression of BCAT1 correlated with lower intracellular BCKAs and glutamate concentrations [18^▪▪^].

Mayers *et al.*[12^▪^] provided an elegant demonstration that the same oncogenic event can result in very different BCAA metabolism and suggested that metabolic activity depended on the tissue-of-origin rather than oncogenic mutation. This study focused on mouse models of PDAC and NSCLC, both driven by Kras mutation combined with p53 deletion. Lung-derived tumors actively took up and catabolized BCAAs to BCKAs, whereas pancreas-derived tumors did not. During oncogenic transformation, PDAC cells even appeared to shut down catabolic flux of BCAAs through downregulating expression of several enzymes in the BCAA catabolic pathway, including BCAT2. In the NSCLC, nitrogen derived from BCAA deamination was used to support biosynthesis of nonessential amino acids and nucleotides [12^▪^] (Table 1).

However, other reports provided evidence that the genetic mutations can also influence how BCAA metabolism impacts cancer progression. Although in the above example of PDAC, BCAA metabolism was suppressed, BCAA catabolism via BCAT2 was recently found to play an important role in PDAC, driven by the chr18q21 chromosomal deletion [11]. Chromosomal region 18q21 is commonly deleted in solid tumors and can impact the expression of many housekeeping genes, including the *mitochondrial malic enzyme 2* (*ME2*). In this study, knockdown of BCAT2 in ME2-deficient PDAC cell lines inhibited colony formation, which could be rescued by nucleotide supplementation, suggesting BCAAs to be an important nitrogen source for nucleotide biosynthesis in this cancer type (Table 1). By contrast, carbon from BCAAs could not be detected in TCA cycle metabolites by metabolic flux analysis, suggesting BCAAs were not a major source of energy in this cell type [11]. Combined, these data suggest that cancer BCAA requirements are dependent on the tissue-of-origin as well as the genetic mutation.

Thus, BCAA metabolism directly influences cancer growth, but different metabolic states are expected based on the cancer type, the genetic mutation, and/or tumor microenvironment in a complex relationship that needs to be addressed in future studies.

## TARGETING BRANCHED-CHAIN AMINO ACID METABOLISM IN CANCER CLINICAL TRIALS

### Recent clinical studies

Likely due to the ease of administration, numerous studies have investigated the consequences of BCAA supplementation on disease progression in clinical trials [35–40]. Although clinical trials investigating BCAAs in different cancers are currently ongoing, most recently published clinical studies involving BCAA in cancer treatments have focused on BCAA supplementation in liver disease and its progression to liver carcinoma [35–38]. Nojiri *et al.*[35] investigated the consequences of BCAA supplementation following radiative ablation of hepatocellular carcinoma in a study involving 51 patients. Several statistically significant differences were observed between the BCAA supplement patients and control group. Importantly, event-free survival was increased, whereas complications were reduced, suggesting BCAA supplementation may be efficacious in this patent population. In a second clinical study, involving BCAA supplementation in hepatocellular carcinoma, Shiozawa *et al.*[39] found that in a study involving 77 patients, BCAA supplementation could also improve patient outcomes. These findings were also corroborated with a third recent clinical observational study, involving 307 patients, which also found that BCAA supplementation benefited patients with advanced liver disease [38].

In addition to clinical trials involving hepatocellular carcinoma, the value of *BCAT1* as a diagnostic marker was recently tested in patients with colorectal cancer, alongside the *ikaros family zinc finger 1* (*IKZF1*) gene [41,42]. Cell-free circulating methylated DNAs of *BCAT1* and *IKZF1* were monitored in patient's blood of nearly 3500 patients scheduled for colonoscopy. The *BCAT1/IKZF1* blood test was found to be 75% positive for recurrences, which points toward its utility in patients with remission [41]. However, further clinical studies are necessary to determine the broader diagnostic value of *BCAT1* status in different cancers.

### Future prospects for therapeutic targeting of branched-chain amino acid metabolism

As described above, several recent studies have found BCAT1 overexpression to be associated with cancer growth and the activity of BCAT1 to be oncogenic [18^▪▪^,^29^]. These studies suggest the prospect of using BCAT1 to develop targeted cancer therapies. Indeed, the fact that *Bcat1*-knockout mice are viable [13,43] suggests there may be a good therapeutic window for targeting BCAT1-dependent cancers.

An alternative approach was recently suggested by Taya *et al.*[44], based on their findings that hematopoietic stem cells (HSCs) required the BCAA valine. HSCs are important for homeostasis of the adult hematopoietic system and are used clinically in HSC transplantation, a curative treatment for a range of hematological diseases including leukemias. For donor HSCs to engraft, recipients must normally undergo irradiation or chemotherapy. Taya *et al.*[44] found that dietary depletion of valine could be used to condition the bone marrow and afford donor HSC engraftment. These findings open up the prospect of metabolic condition regimens based on BCAA modulation.

## CONCLUSION

The past few years of in-depth research on BCAA metabolism in cancer has provided strong evidence for the essential role of BCAAs in tumor progression and has clearly established *BCAT1* as an important prognostic cancer marker. Moreover, BCAA supplementation and *BCAT1* status were tested in clinical trials for hepatocellular carcinoma and colorectal cancer. However, the recent research also revealed a complex addiction of cancer cells to BCAA metabolites, which appear dependent on both the tissue-of-origin and the cancer genetics. This heterogeneous reliance of cancer cells on BCAAs needs to be addressed with future studies so that therapeutic approaches aiming to target BCAA metabolism in cancer can be successfully developed.

## Acknowledgements

None

### Financial support and sponsorship

A.C.W. is supported by Bloodwise (15050) and the National Institutes of Health National Center for Advancing Translational Science Clinical and Translational Science Award (UL1 TR001085). The content is solely the responsibility of the authors and does not necessarily represent the official views of the NIH.

### Conflicts of interest

There are no conflicts of interest.

## REFERENCES AND RECOMMENDED READING

Papers of particular interest, published within the annual period of review, have been highlighted as:

▪ of special interest
▪▪ of outstanding interest

## Figures and Tables

**Table 1:** Cancer type-dependent expression of branched-chain aminotransferase and their downstream effects

Cancer type	BCAT expression	Upstream regulators of BCAT	Metabolites (cancer tissue)	Metabolites (plasma)	Downstream targets of BCAT	Downstream effects	References
Glioblastoma	BCAT1 overexpression in IDH^wt^ BCAT1 epigenetic silencing in IDH^mut^ glioblastomas	IDH^mut^-dependent silencing of BCAT1	In *Bcat1* knockdown:Increased BCAAs Decreased Glu excretion	N/A	*Bcat1* knockdown led to:HADH suppression	*Bcat1* suppression led to:Smaller tumors in mice	[10]
PDAC	ME3 dependent suppression of BCAT2Low BCAT1 expressionHigh BCAT2 expression	ME3, AMPK and SREBP1 regulate BCAT2N/A	In ME3 depleted PATU8988T cells:Increased BCAAsDecreased BCAA uptakeSlightly decreased BCAAsIncreased citrate	N/AIncreased BCAAs	BCAT2-dependent nucleotide biosynthesisN/A	*Bcat2* overexpression led to:Aggressive tumor growth in mice*Bcat1* suppression led to:No change in tumor growth	[11,12^▪^]
NSCLC	High BCAT1 expressionHigh BCAT2 expression	N/A	High BCAAsHigh Glu + GlnHigh nucleotides	Decreased BCAAs	N/A	*Bcat1* suppression led to:Impaired ability to form tumors in mice	[12^▪^]
Ovarian cancer	High BCAT1 expression in EOC	Epigenetic hypomethylation of BCAT1Activation of BCAT1 via c-Myc	*BCAT1* knockdown in SKOV3 cells led to:Low Leu and IleLow sphingolipidsLow glycerophospholipid	N/A	*Bcat1* knockdown led to:suppression of IDH1, IDH2, AKR1C1, and PHGDH	*Bcat1* suppression led to:No effect on tumor burden Increased survival rate in mice	[15]
Breast cancer	High BCAT1 expression in unspecified breast cancer tissuesHigh BCAT1 expression in:ERα^−^, HER2, and TNBC	N/AEpigenetic hypomethylation of BCAT1 in ERα^−^	High BCAAs*BCAT1* knockdown in TamR8 and MDA-MB231 led to: High Glu, Ala, Pro, and BCAAs	High BCAAsLow Glu, GlnN/A	*BCAT1* knockdown led to:Inhibition of mTOR Suppression of *PGC-1a, NRF-1, TFAM, SOD, Catalase, and Gpx1*p27^kip1^ (inhibition) by BCAT1	*BCAT1* overexpression in MCF-7 and T47D led to:Increased growth and colony formation*Bcat1* suppression led to:Decreased tumor size in miceReduced migration and invasion	[16,30]
Liver cancer	High expression in HCC	Activation of BCAT1 via c-Myc	N/A	N/A	*Bcat1* knockdown led to:Suppression of G2/M phaseLC3A/B, p62 (inhibition)	*Bcat1* overexpression led to:Accelerated tumor growth Increased tumor size in mice	[17]
CML	High BCAT1 expression in BC-CML, AML	MSI2 binds BCAT1mRNA	In *Bcat1* knockdown: Low BCAAsLow GluNo change in BCAA uptake	N/A	mTOR (activation)	*Bcat1* and *BCR-ABL1* overexpression in HSPCs led to:Splenomegaly and increased mortality in mice	[18^▪▪^]

AKR1C1, aldo-keto reductase family 1 member C1; Ala, alanine; AML, acute myeloid leukemia; AMPK, AMP-dependent protein kinase; BCAA, branched-chain amino acid; BCAT1, branched-chain aminotransferase 1; BC-CML, blast crisis of chronic myeloid leukemia; EOC, epithelial ovarian cancer; ERα^−^, estrogen receptor negative breast cancer; Gln, glutamine; Glu, glutamate; Gpx1, glutathione peroxidase 1; HADH, hydroxyacyl-CoA dehydrogenase; HCC, hepatocellular carcinoma; HER2, human epidermal growth factor receptor 2 triggered breast cancer; HSPC, hematopoietic stem and progenitor cell; IDH^mut^, tissue specific knock-in of isocitrate dehydrogenase; IDH^wt^, wild-type isocitrate dehydrogenase; Ile, isoleucine; LC3A/B, autophagy marker light chain 3, isoforms A and B; Leu, leucine; MCF-7, breast cancer cell line; MDA-MB231, human breast adenocarcinoma cell line; ME3,malic enzyme 3; MSI2, musashi RNA binding protein 2; mTOR, mammalian target of rapamycin; NRF-1, nuclear respiratory factor 1; NSCLC, nonsmall cell lung cancer; p62, ubiquitin-binding scaffold protein 62; PDAC, pancreatic ductal adenocarcinoma; PGC-1a, peroxisome proliferator-activated receptor-gamma coactivator 1alpha; PHGDH, phosphoglycerate dehydrogenase; SKOV3, ovarian carcinoma cell line; SOD, superoxide dismutase; SREBP-1, sterol regulatory element-binding protein 1; T47D, human breast cancer cell line; TFAM, mitochondrial transcription factor A; TNBC, triple negative breast cancer.
